# Sex-biased chromatin and regulatory cross-talk between sex chromosomes, autosomes, and mitochondria

**DOI:** 10.1186/2042-6410-5-2

**Published:** 2014-01-15

**Authors:** Katherine Silkaitis, Bernardo Lemos

**Affiliations:** 1 Program in Molecular and Integrative Physiological Sciences, Department of Environmental Health, Harvard School of Public Health, 665 Huntington Avenue, Boston, MA 02115-6021, USA

**Keywords:** Gene expression regulation, Drosophila, Sexual dimorphisms, Sex chromosomes, Heterochromatin, Y chromosome, X chromosome, Sex difference

## Abstract

Several autoimmune and neurological diseases exhibit a sex bias, but discerning the causes and mechanisms of these biases has been challenging. Sex differences begin to manifest themselves in early embryonic development, and gonadal differentiation further bifurcates the male and female phenotypes. Even at this early stage, however, there is evidence that males and females respond to environmental stimuli differently, and the divergent phenotypic responses may have consequences later in life. The effect of prenatal nutrient restriction illustrates this point, as adult women exposed to prenatal restrictions exhibited increased risk factors of cardiovascular disease, while men exposed to the same condition did not. Recent research has examined the roles of sex-specific genes, hormones, chromosomes, and the interactions among them in mediating sex-biased phenotypes. Such research has identified testosterone, for example, as a possible protective agent against autoimmune disorders and an XX chromosome complement as a susceptibility factor in murine models of lupus and multiple sclerosis. Sex-biased chromatin is an additional and likely important component. Research suggesting a role for X and Y chromosome heterochromatin in regulating epigenetic states of autosomes has highlighted unorthodox mechanisms of gene regulation. The crosstalk between the Y chromosomes and autosomes may be further mediated by the mitochondria. The organelles have solely maternal transmission and exert differential effects on males and females. Altogether, research supports the notion that the interaction between sex-biased elements might exert novel regulatory functions in the genome and contribute to sex-specific susceptibilities to autoimmune and neurological diseases.

## Review

### Introduction

Sexual dimorphisms in morbidity, mortality, pathology, disease progression, and phenotypic expression have been a matter of abundant research as well as neglect. Studies have documented variable incidence of infection in male and female children [1], disproportionate female susceptibility to immune diseases [2,3], greater risk of mental illness and overall mortality in males [4], variable cancer rates between the sexes [5,6], sex-specific risk for stroke and diseases of aging [7], and unique phenotypic dynamics of sex-biased traits [8,9]. Expectations from population genetic theories highlight the likelihood of such sexual dimorphisms due to transmission biases of the Y chromosome and mitochondria, as well as representation bias of the X chromosome in the sexes [10-13]. Crudely defined molecular mechanisms, however, have prevented a better understanding of genetic variants mediating sexually dimorphic expression and the extent and functional consequences of sex differences is often overlooked. Drug treatment regimen and dosage, for example, typically do not distinguish between men and women [14] despite evidence of pharmacokinetic and pharmacodynamic differences between the sexes [14,15].

Multiple factors contribute to this differential disease susceptibility, including sex hormones and the type and number of sex chromosomes in a genotype. Hormonal fluctuations during pregnancy influence the course and duration of some autoimmune diseases, exacerbating symptoms of systemic lupus erythematosus (SLE) and ameliorating those of rheumatoid arthritis (RA) and multiple sclerosis (MS). In RA and MS, however, while relapse rates decrease during the third trimester, they increase postpartum when hormone levels return to normal [14,16]. Meanwhile, sex chromosome complement, which includes the number and type of sex chromosomes and their genes, is a risk factor in obesity [17,18] and has been implicated in autoimmune susceptibility [19,20]. Y chromosome genetic variation in British men has been associated with blood pressure and total cholesterol levels. One Y chromosome haplogroup in particular is associated with a 50% increased risk of coronary artery disease in men of European ancestry, independent of all other risk factors. Macrophages from men of this haplogroup also display down-regulation of adaptive immunity and up-regulation of inflammatory response pathways [21].

Mutations, deletions, and translocations involving the X chromosome have also been linked to disease phenotypes with a sex bias, including mutations in the *WAS* gene that cause Wiskott-Aldrich syndrome and mutations in *IL2RG* that cause X-linked recessive severe combined immunodeficiency syndrome [22]. While less common, there are also examples of Y-linked immunodeficiencies in mice. The Y-linked autoimmune acceleration (*Yaa*) locus in male mice contains a translocation that includes *toll-like receptor 7* from the X chromosome and contributes to a severe lupus-like phenotype in some mice strains [23]. A recently characterized mouse strain exhibiting Y-linked hereditary B and NK cell deficiencies also highlights the potential for a direct Y chromosome contribution to some autoimmune disorders [24].

Gonadal secretions are essential for triggering and maintaining sexual dimorphisms. Sex determination and sex-specific phenotypes, however, do not spring exclusively from the gonads. Sex differences in embryonic development before gonadal differentiation, observations of sex chromosome-dependent neural and behavioral phenotypes, and the expression of sex chromosome-dependent long non-coding RNAs like *Xist* might all emerge from differences in sex chromosome complement apart from hormonal differences. Sex chromosome factors can include specific genes on the X and Y chromosomes, the ratio of X and Y chromosomes to autosomes, and novel mechanisms emerging from genome-wide gene regulation by sex chromosomes. These factors may play key roles in sex-specific disease susceptibility.

While research identifying loci contributing to sex-biased phenotypes has helped discern disease mechanisms and improve susceptibility assessment in populations and individuals, there are few sex-biased diseases that follow a simple Mendelian inheritance pattern [25]. Similarly, research focusing solely on sex chromosomes, sex-specific hormones, or sex-biased tissues supplies partial answers, but does not fully explain the causes of sex-biased disease and phenotypic expression. Continued attention to sex, environment, and genotype within an integrative framework might contribute a better understanding of the variable penetrance and expressivity of naturally occurring genetic variants and the role of environmental factors in modulating the manifestation of these variants between the sexes.

### Mammalian sex determination

While many autosomal and X-linked genes are dimorphically expressed to yield male and female phenotypes, some sex chromosome-linked genes are expressed solely in one sex or the other and which have essential roles in sex determination. The *sex-determining region Y* (*Sry*) gene is required for testis development in therian mammals. In the absence of sufficient levels of Sry, the gonadal ridge differentiates into ovaries and produces a female phenotype [26-29]. Sry appears to operate primarily as a transcription factor in both gonadal and non-gonadal tissues. In the gonads, Sry binds to the enhancer region of the *SRY-related HMG box protein 9* (*SOX9*) gene, which is essential for inducing Sertoli cells, the primary cell type in the testes, to secrete anti-Müllerian hormone [28,30]. The cascade eliminates the Müllerian ducts, which would otherwise develop into the oviduct and uterus [28,30]. Sry is also notably important in the brain [31,32]. In male adult mice, Sry is present in the substantia nigra, and in rats, *Sry* down-regulation causes a decrease in tyrosine hydroxylase expression and impairs motor activity [33]. The X chromosome gene *monoamine oxidase A* (MAO A), which deaminates monoamine neurotransmitters such as serotonin, is a target of Sry. MAO A plays a critical role in brain development and function, and its abnormal activity has been suggested in sex-biased neurological disorders, such as autism, depression, and attention deficit hyperactivity disorder [34]. Sry may also contribute to the sex bias in Parkinson's disease and schizophrenia, as it might modulate catecholamine synthesis and metabolism in the human male midbrain [35].

### X chromosome inactivation

Another sex-specific gene that might have implications for sex-biased phenotypes in mammals is *Xist*, which codes for a long non-coding RNA whose expression is limited to females. Transcription of *Xist* initiates the inactivation of one X chromosome and leads to equitable expression of X-linked genes in the soma of XY males and XX females. The process begins in the XX zygotes soon after fertilization, when the *Xist* transcript physically coats the X chromosome in *cis* and recruits protein complexes to transcriptionally inactivate the chromosome [36-39]. In mice, there are two distinct stages of X chromosome inactivation (XCI). First, imprinted X inactivation causes the paternal X chromosome to become silenced in early embryogenesis. The second stage of XCI occurs around the time of implantation in the late blastocyst. Cells in the inner cell mass, which will become the fetus, reactivate the imprinted paternal X and subsequently undergo random inactivation of either the maternal or paternal X chromosome. Cells outside the inner cell mass, such as those destined to become either the yolk sac or placenta, retain their paternal X imprinting [36-39].

The process of XCI, however, does not completely eliminate gene expression differences caused by the presence of two X chromosomes. Murine *Xist* is expressed from the two- and four-cell stage onward, but the first cytological signs of XCI are not present until about the 50-cell stage [40]. Similarly, following the early blastocyst re-activation of the paternally imprinted X, there is a period before random inactivation during which females have two active X chromosomes [41]. Finally, after random XCI, some imbalances in gene expression between males and females remain: not all genes in the inactive X chromosome (Xi) are transcriptionally inactive, and not all females express the same number of these Xi escapee genes, nor are the Xi escapees expressed at the same levels [42]. *In vitro*, about 15% of human X-linked genes and about 3% of mouse X-linked genes are expressed on both X chromosomes; an additional 10% of human X-linked genes show variable patterns of XCI [42,43]. Women, but not men, with lupus demonstrate increased expression of X-linked genes, possibly from demethylated regions of the Xi, which may help explain the differential susceptibility of women (and XXY Klinefelter men) to the disease [44]. It should also be noted, however, that other autoimmune diseases such as primary biliary cirrhosis are characterized by haploinsufficiency of X-linked genes [45].

The facultative heterochromatin of the Xi results in females that are a mosaic of two genotypes, depending on whether the paternal or maternal X chromosome was inactivated. This heterochromatin, already exclusive to females, might vary based on whether the Xi was inherited maternally or paternally. Such parent-of-origin imprints have been shown to influence development. Murine XO females with a paternal sex chromosome have delayed prenatal development and are smaller than XX embryos, which are smaller than XY embryos. However, murine XO females with a maternally derived X are significantly larger than their paternal XO counterparts and are equivalent in size to XY embryos [46]. Differences in cognitive function in humans between XO females with a paternal versus maternal X may also be explained by imprinted X genes [47]. The variation in imprinted genes, as well as variable Xi escapee expression, implicates not only the role of various alleles in contributing to a female's mosaic phenotype, but also variable expression depending on parental origin.

The prevalence of each X chromosome's activation state may also be non-random. An interesting possibility is that heterozygous females might have the ability to select neutral alleles over disadvantageous alleles in a tissue-specific manner [14]. Female carriers of agammaglobulinemia, an X-linked immune deficiency, exhibit non-random XCI in B cell lymphocytes [48], and female carriers of Wiskott-Aldrich syndrome exhibit non-random inactivation in all blood cell lineages [49]. Skewed XCI has also been suggested to play a role in disease pathogenesis, such as that of systemic sclerosis (SSc) [50], and it has been documented in breast and ovarian cancers [51,52].

**Figure 1:**
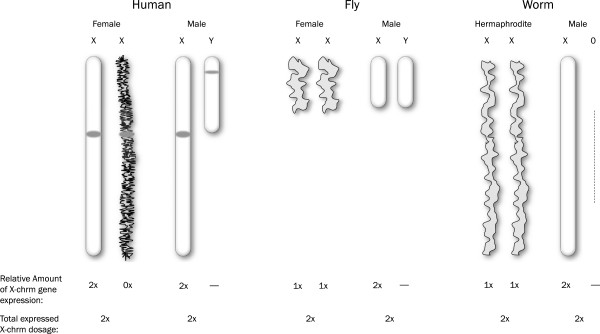
**Dosage compensation mechanisms in human (*Homo sapiens*), fly (*Drosophila melanogaster*), and worm (*C. elegans*).** X chromosome dosage needs to be equalized between the sexes and relative to the autosomes. In humans, females with two X chromosomes undergo X inactivation of one chromosome; the remaining active X up-regulates its genes twofold. In flies, both female X chromosomes are active; male X-linked genes are up-regulated twofold. In worms, which utilize a hermaphrodite/male sex determination pathway, hermaphrodites express X-linked genes at half the rate of males, with both genotypes expressing two times the amount of X-linked genes [39,53]. Chromosomes are not drawn to scale.

The relevance of dosage compensation is illustrated by the unique strategies that have independently evolved to compensate for sex chromosome imbalance. For instance, in *Caenorhabditis elegans*, the XX hermaphrodite expresses genes from both X chromosomes at half the rate as XO males to account for dosage compensation. *Drosophila* males, meanwhile, express X chromosome genes at twice the rate as females, and mammalian females use X inactivation as a dosage compensation mechanism [39,53] (Figure 1).

### Sex chromosome dosage

Some disease phenotypes have been linked to sex chromosome type and number, independent of gonadal secretions. Research in mice has demonstrated a role for the number of X chromosomes in mediating variable susceptibility to adiposity, independent of the presence of a Y chromosome [18]. Additionally, an XX complement in mice, independent of gonadal sex, can increase the risk of lupus and experimental autoimmune encephalomyelitis (EAE; a mouse model of MS) when compared to an XY complement independent of gonadal sex [20,54]. An association between X chromosome number and SLE susceptibility in humans has also been observed: while SLE is more prevalent in women compared to men, the increased prevalence in prepubescent and postmenopausal women precludes a strictly hormonal role. Furthermore, XX females and XXY Klinefelter males display a similar risk, while XO Turner females display decreased disease prevalence [44]. Abnormal karyotypes have been associated with other autoimmune diseases, as well. Men with autoimmune thyroiditis or primary biliary cirrhosis (diseases characterized by female preponderance) display an increased incidence of Y chromosome loss in peripheral blood cells, and women with primary biliary cirrhosis display increased rates of X monosomy [45,55].

The number of X and/or Y chromosomes in mammals might also exert control over the epigenetic cellular machinery, although specific functional consequences on disease dimorphisms are yet to be observed. Embryonic stem (ES) cells with an XX complement, for example, display reduced DNA methylation compared to either XY or XO ES cells [56]. DNA methylation of imprinted alleles in germ cells is influenced by sex chromosome complement as well as the gonadal sex of the embryo [57]. Furthermore, the histone demethylase Kdm3a appears to modulate the level of *Sry* expression in mice. Males with a homozygous *Kdm3a* deletion exhibited frequent partial or full male-to-female sex reversal, some of which were fertile, while females lacking *Kdm3a* underwent normal sex differentiation and were fertile [58].

**Figure 2:**
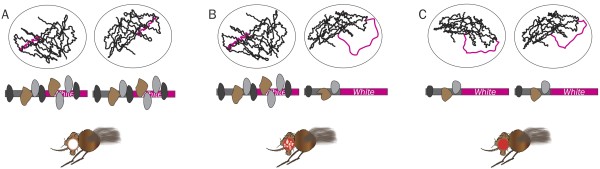
**Position-effect variegation in *Drosophila*.** The stochastic expansion of heterochromatin proteins in each cell can cause a variegated eye color phenotype in *Drosophila*. At the top of each figure is a representation of DNA within a cell, the middle is a representation of the location of heterochromatic proteins within a locus, and at the bottom is the observed eye color phenotype. **(A)** A fly whose cells contain the *white* gene located exclusively in heterochromatin, and thus inaccessible for transcription, will have *white* eyes devoid of red pigment. **(B)** A combination of cells with the *white* gene located in heterochromatin and cells with the *white* gene located in euchromatin, and thus available for transcription, will have a mottled phenotype with some cells producing red pigment and some cells producing no pigment. **(C)** A fly whose cells contain the *white* gene located exclusively in euchromatin will have fully pigmented red eyes.

Sex chromosome complement can influence position-effect variegation (PEV), an epigenetic phenomenon documented in organisms as diverse as yeast, fruit flies, and mammals (Figure 2). PEV occurs when a gene located near a euchromatin-heterochromatin border is randomly silenced or expressed due to the stochastic spreading or contracting of heterochromatin. It was first documented in *Drosophila* in 1930 [59], when a chromosomal translocation moved the *white* gene to a location near heterochromatin. The gene is required for the synthesis of the red pigment in the fly eye, and expansion of heterochromatin causes a mottled-eye phenotype comprising patches of white and red (wild-type) cells. In a mouse model of PEV, males had a greater propensity to silence a human *CD2* reporter transgene than did females. The extent of silencing appeared determined by sex chromosome complement independent of gonadal sex [60].

Finally, evidence suggests that prior to differentiation of the gonads and production of sex hormones, male pre-implantation embryos are larger than those of females [61,62], and some genes, located on both autosomes and sex chromosomes, are already differentially expressed between the two sexes at that stage [63,64]. While sex-determining genes like *Sry* can account for gonad-dependent differences, triggers for pre-gonadal differences are less well defined. The dosage of sex chromosomes might be relevant, as it has a documented influence on sex determination in some species. The ratio of X chromosomes to autosomes determines gonadal sex in fruit flies (*Drosophila*); although the Y chromosome contains genes essential for spermatogenesis, it is not involved in sex determination. Accordingly, XXY genotypes are functionally and phenotypically female, while XO genotypes are sterile males [65]. The nematode *C. elegans* also senses the X/autosome ratio: XX genotypes develop into self-fertilizing hermaphrodites, while XO genotypes develop into males. Some conditions, however, may prompt cross-fertilized XX embryos to lose the paternal X chromosome and develop as XO males [66]. In eutherian mammals, the presence of the Y chromosome gene *Sry* will result in the development of the male gonadal phenotype regardless of how many X chromosomes are present [67]. As a result, an XXY genotype is phenotypically male in mice and humans. Nevertheless, syndromes of X and Y chromosome mono- or polysomies indicate that sex chromosome dosage causes phenotypic variations in humans (Table 1).

**Table 1:** **Sex chromosome dosage and phenotypes in humans and** ***D. melanogaster***

**Genotype**	**Human gonadal sex**	**Human phenotype**	**Fly gonadal sex**	**Fly phenotype**
XO	Female	Turner female^a^	Male	Sterile male
XX	Female	Female	Female	Female
XY	Male	Male	Male	Male
XXY	Male	Klinefelter male^b^	Female	Healthy female
XYY	Male	Slightly atypical male^c^	Male	Lesser characterized male
XXX	Female	Slightly atypical female^d^	Female	Metafemale^e^

‘Male’ and ‘female’ designations are based on gonadal sex. ^a^XO (Turner) females have female external genitals but often have non-functioning ovaries, lack a menstrual cycle, and are sterile. Prevalence is estimated to be 1 per 2,000 live-born females [68]. ^b^XXY (Klinefelter) males have male genitals but are often sterile and hypogonadic. They may display a range of female secondary characteristics, including enlarged breasts and small or undescended testes. Prevalence is estimated to be 1 per 658 live-born males [69]. ^c^Prevalence of XYY males is estimated to be 1 in 1,000, but approximately 85% are never diagnosed. The phenotype commonly includes tall stature, macroenchephaly, macroorchidism, decreased muscle tone, and an increase in autistic spectrum disorder. Some may be at risk of reduced fertility [70]. ^d^Triple X syndrome in females has a variable phenotype, and XXX females will often not display any abnormalities. The prevalence is about 1 per 1,000 female births [71]. ^e^Metafemale *Drosophila* are often sterile and can display narrowed abdomens, wing abnormalities, irregular eye facets, and/or malformed legs. The observed frequency in adults is less that 1%, and viability post-eclosion is limited [72-75].

### Hormonal and sex chromosome interactions

Sex chromosomes and their genes contribute to differential disease susceptibility, but there might also be interactions between sex hormones, sex chromosomes, and the autosomal background. The contribution of sex-specific hormones and sex chromosomes to disease states can be disentangled in a number of ways. The four-core genotypes (FCG) mouse model, in which gonadal sex is independent of sex chromosome complement, is one that has been successfully used. This model was created by combining a *Sry* deletion on the Y chromosome [76] with the insertion of a functional *Sry* transgene onto an autosome. An XX^*Sry*+^ genotype with the autosomal transgene develops testes and is a gonadal male; likewise, an XY^*Sry*-^ genotype lacks *Sry* and develops ovaries to become a gonadal female. The model produces four genotypes, with two genotypes per sex: XX^*Sry*-^ and XY^*Sry*-^ mice are gonadal females lacking the autosomal *Sry* transgene, while XY^*Sry*+^ and XX^*Sry*+^ are gonadal males with the autosomal *Sry* transgene. Here, we use shorthand notation for these genotypes: XXF and XYF for gonadal females, and XYM and XXM for gonadal males. Hence, sex chromosome complement can be studied independent of gonadal secretions initiated by *Sry*, and the interaction between gonadal sex and sex chromosome complement can be observed. This model has yielded insight into the relevance of sex chromosomes to sexual dimorphisms in autoimmune disease, hypertension, neural tube closure defects, and adiposity, among others. For example, sex chromosome complement, independent of hormonal effects, has been implicated in causing differential expression of genes coding for proteins such as calbindin, prodynorphin, and nitric oxide synthase in the brain, and differential expression of two histone demethylases in neurons. It also plays a role in sex differences in aggression, habit formation, and parenting behavior (reviewed in [4,26]). The FCG model also demonstrated a role for *Sry* in regulating autosomal gene expression (e.g., [60]). The regulation may be due to a direct transcriptional role of *Sry* or it may be mediated by sex hormones; the latter is supported by research indicating that XY complement-induced differences in immune response might be suppressed in the presence of testosterone [77]. Finally, cellular models have also contributed to discerning the relative roles of sex hormones and chromosomes in sexual dimorphisms. In one study, Penaloza et al. [78] harvested cells from male and female mice at embryonic stages before and after gonadal differentiation. The data suggested that sex chromosome complement underlies the differential sensitivity of male and female embryonic cells to some stressors, with the introduction of hormonal secretions functioning as a modifier of those differences [78].

### Sex chromosome interactions with autosomes and mitochondria

While the FCG model has helped separate the effects of sex chromosomes versus sex hormones, it has also illuminated sex biases that are partially dependent on genetic background. XX mice face increased susceptibility to adiposity in one strain of mice, while research in another strain suggests that the presence of two sex chromosomes (either X or Y) might be responsible for changes in body weight, body composition, and other metabolic variables [17,18]. Similarly, the contribution of sex chromosomes to EAE and experimental myocarditis susceptibility might be modified by genetic background [79]. The sex reversal caused by the homozygous deletion of *Kdm3a* in mice was also dependent on the genetic origin of the Y chromosome: 14% of C57BL/6 (B6) mice that lacked *Kdm3a* displayed male-to-female sex reversal, whereas the introduction of a CBA Y chromosome in the same *Kdm3a* loss-of-function background resulted in 88% male-to-female sex reversal. The phenomenon might be due in part to the lower levels of Sry in mice with a CBA Y chromosome relative to mice with a B6 Y chromosome, suggesting that CBA mice might already have Sry levels closer to the minimum threshold required for inducing the male development pathway [58].

Interactions between the X chromosome and autosomes might help explain the variable susceptibility of women to autoimmune diseases. Females have an increased prevalence of autoimmune diseases including SLE, RA, MS, SSc, primary biliary cirrhosis, Hashimoto's thyroiditis, and pernicious anemia [2]. Some of the risk factors are genetic, as concordance studies in twins demonstrate, but incomplete concordance also demonstrates the relevance of non-genetic factors [80]. Autoimmune diseases vary greatly in their mechanisms, penetrance, response to treatments (which can include sex hormone therapy), and underlying genetic causes. An interesting possibility is that increased female susceptibility might emerge from polygenic autosomal factors on a permissive X chromosome background and in a permissive environment. The increased prevalence of RA in urban Senegalese populations compared to their rural counterparts suggests environmental triggers in otherwise healthy but genetically susceptible population [81]. Similarly, the TRAF1/C5 polymorphism has been implicated in susceptibility to RA in a North African population and with susceptibility to SLE in a European population [82]. The variation might be attributable, in part, to genetic background interactions and/or environmental triggers. Finally, accumulating evidence suggest that environmental agents may influence the development of lupus by inhibiting T cell DNA methylation [44].

One hypothesis that might partially explain the origin of some male-biased diseases rests in the maternal transmission of the mitochondrial genome. The asymmetrical transmission precludes the purging of mutations harmful to males if they are beneficial, neutral, or only slightly disadvantageous for females. This ‘mother’s curse’ was implicated in reduced sperm function and fertility in males with mtDNA mutations, while female fertility was unaffected [83]. The curse might also have further repercussions on health and aging. Genetic variation in *D. melanogaster* mitochondrial genomes appeared to affect male-specific patterns of aging, while females remained unaffected [84]. Similar research in *D. melanogaster* documented significant differential gene expression in males with mtDNA introgressions (more than 8% of tested genes among five introgressions), yet very few differentially expressed genes in females of the same lines (about 0.06%) [11]. Evidence of coevolution of the male mitochondrial genome with the nuclear genome is abundant [10,85,86]. Males carrying one mtDNA haplotype might be sterile when introgressed into an isogenic background, but fertile when expressed in its coevolved genetic background [11]. Finally, interspecific cellular hybrids with mismatched nuclear-mitochondria pair display a range of anomalies [87]. These include cellular inviability, which can manifest even if the species donating the mitochondria and the nuclear genome are closely related [87].

### Sex chromatin structure and epigenetic modifications

Evidence of sex chromosome modulation of autosomal gene expression and downstream phenotypes is rapidly accumulating. However, elucidating the genetic elements that mediate sex chromosome interaction with autosomes has lagged and complicated attempts to explain differential responses to nearly identical circumstances. For instance, a specific deletion in the Y chromosome contributes to male infertility in some human populations, but not others [88], suggesting interactions with the genetic background. Clues to the mechanisms for such differential effects might partly lie in the genetic variation of Y chromosomes and possibly in novel regulatory forces exerted by heterochromatic segments of the chromosome.

**Figure 3:**
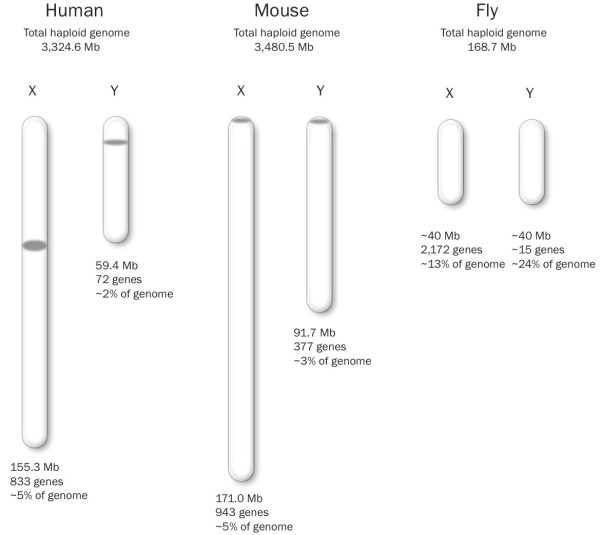
**Relative sizes of the X and Y chromosomes in human (*H. sapiens*), mouse (*Mus musculus*), and fly (*D. melanogaster*).** Drawn to scale. Gene counts are for protein-coding genes and do not reflect the number of copies in multi-copy genes. *H. sapiens* and *M. musculus* data obtained from Ensembl database release 73 [91] and Pertile and Graham [96]. *D. melanogaster* X and Y chromosome size obtained from the literature [89,90]. X chromosome gene count obtained from Ensembl database release 73 [91]. Y chromosome gene count from the literature [89,90].

In *D. melanogaster*, the Y chromosome harbors 15 protein-coding genes and accounts for nearly 25% of male haploid DNA content [89,90]; in contrast, the X chromosome is about the same size and contains more than 2,000 genes [91] (Figure 3). This incongruity occurs because much of the Y chromosome is heterochromatic and comprises transposable elements and other repetitive sequences. Research using Y chromosome introgressions in isogenic and reciprocal genetic backgrounds revealed that the Y chromosome can regulate response to temperature, fertility, spermatogenesis, and fitness, as well as the expression of hundreds of X-linked and autosomal genes [92-95].

Differential gene regulation and expression, however, are dependent upon the autosomal and X chromosome background. When comparing *D. melanogaster* populations from temperate and tropical climates, Y chromosome origin accounted for about 50% of the difference in susceptibility to heat-induced sterility [93]. Similarly, consomic strains generated with Y chromosomes in various genetic backgrounds revealed that fitness was dependent on the interaction between variable Y chromosomes and the genetic background [95]. Furthermore, increased amounts of Y chromosome DNA in a male fly may cause higher expression of PEV markers [97]. Finally, Y chromosomes of different geographic origins were found to differentially modulate PEV on an identical autosomal background such that some Y chromosome variants suppressed the expansion of heterochromatin, and others enhanced it [98]. These observations may indicate regulatory roles for sex chromosomes in mediating disease susceptibility.

One hypothesis for the mechanism by which a gene-poor chromosome regulates autosomal gene expression is through the sequestration of heterochromatic factors. Differential sequestration of these factors by polymorphic regions of the Y chromosome might explain the modulation of both PEV and genome-wide gene expression. Consistent with this hypothesis is the observation that XXY *D. melanogaster* females with polymorphic Y chromosomes show differential expression of thousands of genes despite not expressing Y-linked proteins [98]. In *D. melanogaster*, the Y chromosome also contains variation in repeat number of the multi-copy rDNA locus, which leads to both differential PEV and global gene expression [99].

In addition to the hypothesis that the Y chromosome serves as a heterochromatic sink, at least two complementary and non-mutually exclusive hypotheses are evident [100]. First, variable Y chromosomes might contribute distinct pools of small RNAs. Second, Y chromosome variation might perturb the spatial arrangement of chromosomes in the nucleus. Genes responsive to the Y chromosome may show restricted nuclear distribution, although the arrangement is bound to be variable across cell types [100]. Interestingly, Branco et al. [101] recently showed that the manifestation of variable Y chromosomes on gene expression requires wild-type function of the heterochromatin protein 1 (HP1). They also observed contrasting effects between testis-specific and somatic gene expression that emerged from the genetic interaction between *HP1* and the Y chromosome. Interestingly, HP1's role in nuclear architecture and its association with nuclear lamin proteins have long been known [102,103]. They raise the possibility that naturally occurring variation in the Y chromosome might modulate nuclear architecture and alter the accessibility of the transcription machinery to specific genes.

In mice, the number of X chromosomes appears to influence PEV and reporter gene expression, whereas a contribution from the Y chromosome is not apparent. This might occur because the mouse Y chromosome represents a much smaller percentage of the haploid male mouse genome (about 3%) than it does in *Drosophila*. Female mice, however, have a heterochromatic inactive X chromosome which appears to play a role in regulating PEV. Male mice show markedly increased PEV silencing of a reporter gene compared to females, but analyses with the FCG model suggest that the phenomenon might be due to X chromosome number rather than presence of a Y chromosome. Accordingly, both XYM and XYF mice showed greater silencing than XXM or XXF. The use of an additional mouse model, in which a modified Y chromosome is attached to an X chromosome to produce XXY males for comparison with XO females, separated the individual effects of the X and Y chromosomes. XXY males created with this model displayed less silencing of the reporter gene than the XO females [60]. Hence, the molecular role of the *Drosophila* Y chromosome as a likely sink for heterochromatin factors might have its mammalian counterpart in the inactive X chromosome [77].

As the regulatory nature of sex chromosomes is likely to revolve around epigenetic mechanisms, it is relevant to note that the differential expression of genes in XX versus XY mice may also be due to Xi escapees, many of which code for chromatin proteins [77]. This observation, combined with variable Xi escapee patterns [42], further supports the notion that sex chromosomes contribute to autosomal and X chromosome gene expression through chromatin remodeling. HP1, a major modifier of PEV in mice and *Drosophila*, provides further evidence of sex bias in chromatin. The protein appears to exert sex-specific gene regulation in *Drosophila*, and deletion causes sex-biased lethality [104]. Interestingly, genes identified as responsive to sex chromosome complement in mammals were enriched for candidates sensitive to HP1 [60].

### Sex chromatin in genotype-by-environment interactions

Sex-biased chromatin states on autosomes (e.g., differentially methylated DNA between the sexes) might emerge from *trans* regulation by sex chromosomes. This genetic interaction might set the stage for additional second-order interactions with the environment and manifest as global gene expression patterns. Maternal nutrient restriction (MNR) during fetal growth may illustrate the potential for environmental modulation with long-term effects. The research emerged in part due to accurate record keeping through the Dutch Hunger Winter, a period during World War II in which the western part of the Netherlands went through official food rations. Health and birth records remained intact in three hospitals, and cohort studies traced the lifelong health and disease trajectories of individuals conceived and born during this time [105]. The studies have provided insight into the developmental origins of health and disease and highlighted possible early origins of sex differences.

Lipid profiles of adults exposed prenatally to famine exhibited sex bias independent of gestational timing: adult women who experienced prenatal nutrient restriction showed elevated total and LDL cholesterol and triglyceride concentrations, risk factors for cardiovascular disease, compared to unexposed women; men did not show such an increase [106]. Furthermore, Tobi et al. [107] compared DNA methylation patterns in 15 genes associated with metabolic and cardiovascular disease in individuals prenatally exposed to famine. The results suggest that the timing of famine exposure might underlie gene expression and methylation differences. Specifically, six loci displayed methylation differences compared to an unexposed same-sex sibling; the association differed by sex in three loci. When eight loci were tested for methylation differences in late-gestational exposure to famine, men displayed methylation differences in two of the three sex-associated loci. Methylation differences included both increases and decreases, and one locus displayed both an increase and decrease depending on timing of famine exposure [107]. Similar sex-specific methylation in response to MNR was observed in sheep. A periconceptual modest reduction of B vitamins resulted in adult phenotypes that included elevated blood pressure, insulin resistance, and obesity. Exposed adult offspring exhibited altered methylation states in 57 of 1,400 CpG islands: 88% of the loci were hypomethylated or unmethylated compared to controls, and 53% of the altered loci were specific to males, while 12% were specific to females [108]. These associations and sex-specific effects are intriguing and might include both causative and correlated epigenetic modifications.

Maternal stress in mice might also influence offspring behavior in a sex-dependent manner. Male offspring exposed to early prenatal stress (E-PS) displayed behavioral changes and depressive-like phenotype as adults [109]. Male mice also showed altered expression levels of stress-responsive proteins: corticotropin-releasing factor expression was increased in the central nucleus of the amygdala, while glucocorticoid receptor expression was decreased in regions of the hippocampus. In the hypothalamus, the corticotropin-releasing factor promoter had reduced levels of DNA methylation and the glucocorticoid receptor promoter had increased methylation; additionally, the corticotropin-releasing factor promoter in DNA isolated from the central nucleus of the amygdala also had reduced methylation. Since the fetal brain is not yet formed at the E-PS period, the long-term, sex-specific epigenetic effects on behavior might be mediated by sex-specific changes in placental gene expression. Placental gene expression analyses in E-PS mothers of males revealed up-regulation of *peroxisome proliferator-activated receptor alpha* (*PPARα*), *insulin-like growth factor binding protein 1* (*IGFBP-1*), *glucose transporter 4* (*GLUT4*), and *hypoxia-inducible factor 3a* (*HIF3a*). However, the placenta from mothers of females showed down-regulated *PPARα*. DNA methylation machinery also varied between male and female embryos and between control and E-PS embryos. The methylation maintenance enzyme DNMT1 was lower in male control compared to female control placentas: E-PS caused no significant change in expression in males, but caused a significant increase in enzyme expression in females [109]. Sex-biased placental response in *PPARα* methylation may also provide a mechanism for the sex-biased disease phenotypes seen in response to MNR. DNA methylation in the *PPARα* promoter decreased in the liver of rats prenatally exposed to a protein-restricted diet. While the promoter methylation decrease was small, from 6.1% to 4.5%, the change corresponds to a 26% decrease compared to controls and accounted for up to 43% of the variance in gene expression of *PPARα*[110]. These results might reveal both the disruption caused by exposure to stress as well as mechanisms of stress protection.

### Sex-biased disease phenotypes responsive to Y chromosome genetic background

Although specific mechanisms and causal networks are poorly defined, sex chromosome and background interactions are likely relevant to human disease states. Sex chromosome-dependent gene expression variation in immune response genes might be one pathway for modulating disease phenotypes. Y-linked regulatory variation, the quantitative effects of polymorphic Y chromosomes on genome-wide gene expression seen in *Drosophila*, may provide clues to key cellular mechanisms with phenotypic consequences. Aside from the disproportionate modulation of genes that code for protein products that localize to the nucleus and which might modify chromatin dynamics, there is a substantial contribution of Y chromosome origin to the differential expression of immune response genes [79,98,111].

Murine disease susceptibility mediated by Y chromosome origin has also been documented in response to challenge with the coxsackievirus B3 (CVB3). Twelve Y chromosome consomic strains were generated on a B6 background, and CVB3-induced mortality in the consomic strains exhibited a continuous distribution. Although sex hormones have been shown to mediate CVB3 susceptibility, the pattern of mortality was found to be independent of prenatal or adult testosterone levels [112]. Thus, the role of the Y chromosome in infectious disease susceptibility may be in part non-hormonal.

EAE is a widely used animal model for studying the pathogenesis of MS. In humans, MS is more prevalent in women, and the ratio of women to men appears to be increasing [113-115]. One reason for the sex bias might be the protective effect of testosterone. EAE in three strains of mice has a similar sex bias as seen in human MS. Investigation using one strain (SJL) documented that castration increased disease susceptibility in male mice, presumably due to the decrease in testosterone upon removal of the male gonads [116]. Castrated males and normal females developed a similar disease course [116]. Castration of male mice similarly increases disease prevalence and susceptibility in models of non-obese diabetes, thyroiditis, and adjuvant arthritis, diseases that have a similar sex bias as MS [3]. Furthermore, testosterone levels are inversely correlated with disease progression in males, peaking after recovery and at the lowest levels during the height of the disease [117]. Nevertheless, in two strains of mice, the sex bias in EAE is reversed (males display increased susceptibility), whereas another strain shows no sex bias (reviewed in [3]). This result echoes earlier murine research in which the effect of androgen removal on EAE was dependent on genetic background [118], and observations that autosomal gene associations with MS susceptibility are often sex-specific in humans [119,120].

Interestingly, genetic variation specifically on the Y chromosome affects EAE susceptibility in male mice [116,121]. While prevalence in SJL mice mimics that of humans, the female-to-male ratio decreases with age, due to the increasing susceptibility of aging male mice. While lower testosterone influences male SJL susceptibility [116], Spach et al. [121] demonstrated a role for the Y chromosome as well. Consomic strains of SJL and B10.S mice were generated with the reciprocal Y chromosome, resulting in SJL.Y^B10.S^ and B10.S.Y^SJL^ strains. While B10.S and B10.S.Y^SJL^ mice displayed similar resistance to EAE, the phenotype of SJL and SJL.Y^B10.S^ mice diverged, and SJL.Y^B10.S^ mice displayed a more severe disease course than the SJL mice [121].

One hypothesis is that copy number variation in the Y chromosome modulates EAE and experimental myocarditis susceptibility. In mouse models, Y chromosome substitution lines show that susceptibility to these diseases is correlated with the number of repeats of the Y-linked genes *Sly* and *Rbmy*[79]. These Y chromosome structural polymorphisms might modulate global gene expression and alternative splicing in a cell-type specific manner that depends on genetic background. A comparison of the mRNA expression in CD4+ T cells between a Y chromosome introgression and its unaltered counterpart revealed 734 differentially expressed transcripts. In the same comparison, 64% of chromatin remodeling genes assayed were differentially expressed, and 3,247 transcripts were alternatively spliced [79].

Evidence of sex chromosome and background interactions in autoimmune disease has also been observed using the FCG model. A sex chromosome effect on EAE was observed in castrated SJL mice: XXM and XXF displayed a more severe disease course than did XYM or XYF. However, when the FCG model was used to investigate sex chromosome effects in a mouse strain that did not display a sex-biased MS phenotype (the C57BL/6 strain), disease outcomes did not differ when comparing either of the XX or XY genotypes. The mouse model for lupus, which is characterized by a 9:1 female-to-male ratio in humans, revealed a sex chromosome effect in the FCG SJL mice [54]. Gonadectomized XX mice of both sexes exhibited greater disease severity and mortality than either of their XY counterparts. In gonadally intact mice, XXF had significantly higher mortality than XYF, whereas neither XYM nor XXM showed significant mortality during the duration of the study [54]. These results suggest the influence of sex chromosomes as well as the protective effects of testosterone in gonadal intact male mice.

Finally, recent research points toward a role for the Y chromosome in regulating cardiac phenotypes, neonatal programming, and chromatin structure in mice [122,123]. Llamas et al. [123] generated consomic strains with a Y chromosome from either a C57BL/6 J (Y^B6^) or A/J (Y^A/J^) strain on a B6 background and noted that cardiomyocytes from mice with a Y^B6^ were larger than those from mice with a Y^A/J^. Increased cardiomyocyte size is a characteristic of cardiomyocyte hypertrophy, a stress response that can be beneficial but is also associated with sudden death and overt heart failure [124]. They noted that the reduced size of B6-Y^A/J^ cardiomyocytes was due to the absence of hypertrophic effects of post-pubertal testosterone on the cells, but that testosterone did cause differential gene expression in the two strains [123]. Additionally, the consomic strains showed differential genomic occupancy of androgen receptors in cardiac chromatin from intact adult mice and in neonatal hearts. B6-Y^B6^ mice displayed signatures of androgen-receptor binding that were significantly enriched for genes related to cardiac morphology [122]. Hence, the data raise the possibility that differences in the manifestation of cardiovascular disease in men and women might be influenced in part by the Y chromosome.

## Conclusions

Sex chromosome dosage, sex chromosome genes, and sex hormones underlie sex-specific phenotypic and sex-biased expressions. Interaction of these factors with the genetic background of autosomes and mitochondria further contributes to sex-biased phenotypes and explains the components of within-sex and between-sex variation. Heterochromatin load on the Y chromosome and on the inactive X chromosome adds another component and possibly new mechanisms to sex chromosome mediation of epigenetic states on autosomes. Both the X and Y chromosomes have been shown to differentially modulate global gene expression, including examples in which the chromosomes play a role in determining susceptibility to murine models of obesity, lupus, MS, and cardiac phenotypes. However, outcomes are often conditional on the genetic background of autosomes and sex chromosomes. Recent models in *Drosophila* and mouse suggest molecular mechanisms of polymorphic sex chromosome action and indicate that phenotypic responses are sensitive to environmental stress. Finally, the interaction of sex chromosomes with the mitochondrial background might be relevant to the emergence of sex-specific chromatin states.

Altogether, the lack of well-parameterized models for how the chromatin of distinct chromosomes interacts and produces perturbations that can be detected as trans-regulatory effects needs broader acknowledgement. Some independent contributions, such as mitochondrial mother's curse in males, are well defined, but mechanisms for the expression of the curse are often less clear. Indeed, a related challenge has been to systemically address the interaction between genetic elements that might have evolved under disparate pressures (e.g., X chromosome, Y chromosome, and mitochondria). We envision that understanding how these genetic elements interact will reveal mechanisms of sex-biased diseases in somatic tissues, which might intersect unique pathways of sex chromosome action.

## Abbreviations

EAE: experimental autoimmune encephalomyelitis (mouse model of multiple sclerosis); FCG: four core genotypes; MNR: maternal nutrient restriction; MS: multiple sclerosis; PEV: position-effect variegation; RA: rheumatoid arthritis; SLE: systemic lupus erythematosus; SSc: systemic sclerosis; Xi: inactive X chromosome.

## Competing interests

The authors declare that they have no competing interests.

## Authors' contributions

KS and BL drafted and wrote the manuscript. Both authors read and approved the final manuscript.

## Authors' information

KS is a doctoral student in Molecular and Integrative Physiological Sciences in the Department of Environmental Health at the Harvard School of Public Health. BL is an Assistant Professor of Environmental Epigenetics at the Harvard School of Public Health.
